# Playing-related musculoskeletal disorders in Malaysian orchestral musicians: a cross-sectional survey

**DOI:** 10.3389/fpubh.2026.1838414

**Published:** 2026-06-03

**Authors:** Yanran Xu, Ahmad Hazwan Ahmad-Shushami, Shafa'atussara Silahudin

**Affiliations:** 1 Academy of Malay Studies, Universiti Malaya, Kuala Lumpur, Malaysia; 2 Department of Sports Medicine, Faculty of Medicine, Universiti Malaya, Kuala Lumpur, Malaysia

**Keywords:** cross-sectional study, Malaysian orchestral musicians, MPIIQM, orchestral musicians, playing-related musculoskeletal disorders (PRMDs)

## Abstract

**Introduction:**

Playing-related musculoskeletal disorders (PRMDs) are common occupational conditions among orchestral musicians. However, data about the prevalence of PRMDs in Southeast Asia—and Malaysia specifically—are limited. This study estimated the prevalence, characteristics, and associated factors of PRMDs, while measuring scores for pain intensity and interference in Malaysian professional orchestral musicians.

**Methods:**

A cross-sectional survey was conducted among professional orchestral musicians in Malaysia using the Musculoskeletal Pain Intensity and Interference Questionnaire for Musicians (MPIIQM). Prevalence rates for PRMDs were calculated for lifetime, 12-month, 1-month, and point prevalence. Anatomical distributions were analyzed by instrument type and gender. Univariable and multivariable binary logistic regression analyses were performed to examine associated factors for PRMDs. Pain interference and pain intensity scores were derived for individuals experiencing PRMDs.

**Results:**

Lifetime prevalence was 84.5% (*n* = 443), while 12-month prevalence was 48.1% (*n* = 252), 1-month prevalence 40.6% (*n* = 213), and point prevalence 39.5% (*n* = 207). Among musicians with PRMDs, the most common regions affected were the neck, shoulders, and upper limbs; 62.3% (*n* = 137) had pain in three or more regions. Mean pain intensity and interference scores were 12.19 ± 1.916 (range 0–40) and 15.26 ± 3.214 (range 0–50), respectively. Female gender, string instrument playing, and younger age are associated with PRMDs.

**Conclusion:**

This study confirms that PRMDs are common in Malaysian professional orchestral musicians. The use of an operational definition and a validated assessment tool allows for more accurate and meaningful estimates of pain prevalence. Female gender, string instrument playing, and younger musicians may be associated with PRMDs. These results support the need for targeted preventive and ergonomic interventions and emphasize the importance of standardized measures in music performing arts medicine.

## Introduction

Previous research has shown that the professional orchestral environment involves substantial physical and psychosocial demands, which have been associated with playing-related musculoskeletal disorders (PRMDs) (1, 2). These demands arise from prolonged practice, repetitive movements, sustained postures, and high requirements for technical precision, often combined with intensive rehearsal schedules and limited recovery time. In addition, organizational and contextual factors—including workload, performance pressure, and psychosocial work environment—may further contribute to the development and persistence of PRMDs in orchestral musicians (1, 2).

Within this context, a lot of epidemiological literature has consistently documented a high prevalence of PRMDs among both professional and student musicians. Contemporary studies report prevalence estimates ranging from approximately 48% to over 90%, depending on the operational definition and measurement approach used (3–6). Specifically, within orchestral populations, prevalence rates have been reported in the range of approximately 55 to 95% (7), indicating that PRMDs represent a widespread occupational health concern in this population. Importantly, recent longitudinal and multicenter research has substantially advanced the understanding of PRMDs by identifying a broader set of prospective risk factors. These include fatigue, changes in physical activity, psychosocial stressors, perfectionism, and occupational workload (8–10).

Despite the large body of research conducted, systematic reviews have highlighted limitations in studies examining the prevalence rates and risk factors for musculoskeletal disorders among professional musicians (5, 6, 11–15). Common methodological issues include the lack of a uniform definition of PRMDs, the use of heterogeneous and often non-validated measurement tools, reliance on cross-sectional designs, limited representation of Asian and non-Western orchestras, and insufficient attention to psychosocial and organizational determinants of musicians’ health. These limitations have contributed to the wide variability in reported prevalence rates, and have impeded meaningful comparisons across studies.

To address such issues in methodology, Berque et al. (16) developed the Musculoskeletal Pain Intensity and Interference Questionnaire for Musicians (MPIIQM), a biopsychosocial self-report tool specifically designed for professional orchestral musicians. This tool provides a standardized operational definition alongside pain intensity and interference scores. The MPIIQM is widely used globally, with validated cross-cultural translations to German (17), Polish (18), South African English (19), and Portuguese (20).

Regarding terminology, one of the most frequently referenced definitions is that of Zaza and Farewell (21), which defines PRMDs as “any pain, weakness, numbness, tingling, or other physical symptom that interferes with your ability to play your instrument at the level you are accustomed to. This definition does not include transient mild aches or pains.” In an attempt to improve this definition, Berque et al. (16), adopted a more broadly encompassing understanding of the issue by shifting the terminology from “disorders” to “pain/problems,” abbreviated as PRMPs. This study uses the term PRMDs, but uses it with a similar understanding as that encompassed by the Berque et al. (16) PRMP framework.

Though considerable progress in relevant research work has occurred in Europe, North America, and other areas of East Asia, there is a surprisingly small body of research that involves Southeast Asian orchestral musicians. This lack of Southeast Asian data limits the global understanding of PRMDs. In Malaysia, professional orchestral musicians operate within a distinct cultural and occupational context; this is shaped by diverse musical traditions and limited musician-specific formalized occupational health frameworks, which may influence both the experience and management of PRMDs.

To fill this research gap, this research will make use of the MPIIQM to establish prevalence rates, levels of pain intensity and interference, and associated factors for PRMDs among Malaysian orchestra musicians. This research is significant as it seeks to provide epidemiological evidence to understand orchestral musicians’ occupational health using a well-designed, globally validated instrument in Malaysia.

## Method

### Definition of PRMDs

In this study, PRMDs were defined operationally as **“**any pain, weakness, numbness, tingling, or other physical symptom that interferes with your ability to play your instrument at the level you accustomed to. This definition does not include transient mild aches or pains” (21). This definition has been widely adopted in epidemiological studies of musicians and is considered a standard approach for enhancing comparability and methodological consistency in prevalence estimation (19, 22–24).

### Study design

A cross-sectional study design was used to investigate the prevalence and associated factors of PRMDs among professional orchestral musicians in Malaysia. The MPIIQM was used for data collection. Ethical approval was obtained from the University of Malaya Research Ethics Committee (UMREC) (Reference Number: UM. TNC(P&I)/UMREC_5225). All participants provided their informed consent before data collection. Participation was voluntary, and data were kept anonymous, with no identifiable personal data collected. All data were stored on a computer accessible only by researchers.

### Participants and samples

A convenience sampling method was used to recruit participants from professional orchestras in Malaysia. The sampling frame included musicians from five orchestras: the Malaysian Philharmonic Orchestra (MPO), the Penang Philharmonic Orchestra (PPO), the National Symphony Orchestra (NSO), the Selangor Symphony Orchestra (SSO), and the Selangor Philharmonic Orchestra (SPO). These five orchestras collectively represent the majority of professional orchestral musicians in Malaysia and reflect different organizational types and performance settings. Study invitations were distributed to eligible musicians via email with a link to an online questionnaire. The sample size was calculated based on point prevalence, using the formula for estimating population proportion (25): $n=\frac{Z^{2}P(1-P)}{d^{2.}}$ .

where n is required sample size, Z is the Z value corresponding to the desired confidence level (95%), P is the expected prevalence, and d is the margin of error. The expected prevalence was set at 36.6% (*p* = 0.366), according to previous reports (22). A 95% confidence level (*α* = 0.05, Z = 1.96) and a marginal error of 5% (d = 0.05) were applied. The minimum calculated sample size was 357 subjects. Considering a 20% attrition rate due to potential invalid responses, the required number of valid completed questionnaires was adjusted to 447. Based on previous reports of similar cross-sectional surveys, the expected response rate was estimated at 40%. Therefore, the distribution of 1,118 online questionnaires was planned among the target population.

Participants were eligible for inclusion if they: (a) were at least 18 years old; (b) were currently engaged as a professional orchestral musician in Malaysia, either full- or part-time; (c) had a minimum of 10 years of instrumental performance experience, applied as a pragmatic indicator of sustained cumulative exposure to instrumental playing; and (d) were able to comprehend and complete the questionnaire in English. English was selected as the survey language, as professional orchestral musicians in Malaysia typically communicate professionally in English. Musicians who were less than 18 years old, were not actively performing in a professional orchestra at the time of data collection, or who did not meet the minimum instrumental experience criteria were excluded from the study.

### Questionnaire and data collection

Data was collected using the MPIIQM, a validated instrument designed to assess PRMDs among professional musicians. The MPIIQM evaluates pain intensity and interference in musical performance. The scale demonstrates high internal consistency, with an overall Cronbach’s alpha of 0.88.

The questionnaire comprises 22 items divided into two sections. The first section includes 12 items collecting demographic information and basic playing-related characteristics, including yes/no questions and total weekly hours of practice. The second section contains 10 items assessing pain experience, with responses on a 0–10 numeric rating scale. Four items measure pain intensity, and five items assess pain interference; this is further subdivided into affective interference (two items) and functional interference (three items). Pain intensity scores were calculated by summing the four intensity items (range 0–40), while pain interference scores were obtained by summing the five interference items (range 0–50). One item uses a body outline diagram for participants to indicate the precise locations of pain.

Access to the orchestras was arranged via orchestra managers, who were only involved in distributing the study invitation and online survey link. To prevent the perception of coercion, the names of participating members were not communicated to the managers, and no reminders were sent via the management. The questionnaire was answered voluntarily online, and participation was entirely anonymous.

### Data analysis

Data was analyzed using IBM SPSS Statistics for Windows, version 27.0 (IBM SPSS Corp., Armonk, NY, USA). Descriptive statistics were used to summarize demographic characteristics and PRMD prevalence and characteristics. Prevalence was calculated for different time frames: lifetime prevalence was calculated as the proportion of participants who reported ever experiencing PRMDs during their professional career; 12-month prevalence was the proportion of participants reporting PRMDs in the past 12 months; 1-month prevalence was the proportion of participants reporting PRMDs in the past month; and point prevalence was the proportion of participants experiencing PRMDs in the last week or at the time of data collection.

Categorical variables were compared between PRMD and non-PRMD groups using chi-squared tests. Continuous variables were compared using independent sample t-tests for normally distributed data and Mann–Whitney U tests for non-normally distributed data. Data normality was assessed using the Shapiro–Wilk test. Variables with *p* < 0.25 in the univariate analysis were entered into a multivariable binary logistic regression model to identify associated factors for PRMDs as recommended by Hosmer et al., (26), to ensure that potentially important variables are not prematurely excluded due to limited statistical power in univariate testing. Statistical significance was set at *p* < 0.05.

## Results

### Participants’ characteristics

A total of 1,118 questionnaires were distributed. A response rate of 51% yielded 570 questionnaires. After excluding 46 incomplete or invalid questionnaires, the final valid sample consisted of 524 questionnaires (Table 1), of which 271 were from male and 253 from female participants. In terms of instruments, 238 participants played string instruments (upper strings, *n* = 148; lower strings, *n* = 90). A total of 294 participants were full-time orchestral musicians, while 230 were part-time.

**Table 1:** Participants’ characteristics.

Categorical variables			*n* (%)	
Gender	Male		271 (51.7)	
	Female		253 (48.3)	
Instrument type	Strings	Upper strings	148 (28.2)	238 (45.4)
		Lower strings	90 (17.1)	
	Woodwinds		125 (23.8)	
	Brass		100 (19.0)	
	Percussion		61 (11.6)	
Employment status	Full-time		294 (56.1)	
	Part-time		230 (43.9)	

Continuous variables	Median (IQR)	Range
Age (years)	38 (34, 41)	26–48
Years of playing instrument	21 (18, 23.75)	11–34
Years in orchestra	19 (15, 24)	10–28
Weekly playing hours (inside orchestra)	19 (16, 23)	13–27
Weekly playing hours (outside orchestra)	9 (7, 11)	5–13

All continuous variables were tested for normality using the Shapiro–Wilk test and showed significant deviation from normal distribution (*p* < 0.05). Therefore, these variables are presented as median with interquartile range (IQR). The median age of participants was 38.0 years (IQR: 34.0–41.0; range: 26–48). The median years of playing experience were 21.0 years (IQR: 18.0–23.75; range: 11–34). The median years of orchestra membership were 19.0 years (IQR: 15.0–24.0; range: 10–28). The median weekly playing time inside the orchestra was 19.0 h (IQR: 16.0–23.0; range: 13–27), and the median weekly playing time outside the orchestra was 9.0 h (IQR: 7.0–11.0; range: 5–13).

### Prevalence rates and characteristics of painful body locations

Among the 524 participants, the lifetime, 12-month, 1-month, and point prevalence of PRMDs were 84.5% (*n* = 443), 48.1% (*n* = 252), 40.6% (*n* = 213), and 39.5% (*n* = 207), respectively.

The MPIIQM first presents participants with the standard PRMD definition (21). Participants were then asked to report the presence of PRMDs across four timeframes: lifetime, 12-month, 1-month, and point (past week) prevalence. Consistent with the MPIIQM’s structure, only participants who reported symptoms within the past month or/and past week proceed to complete the detailed pain intensity and interference subscales and the body diagram. This design choice prioritizes the accuracy of detailed pain characterization by focusing on recent symptoms, thereby reducing recall bias. Based on this criterion, a total of 220 participants reported PRMDs, and 324 participants reported non-PRMDs.

Among the 220 musicians who reported PRMDs, 15 (6.8%) reported pain in a single body location, 68 (30.9%) reported pain in two locations, 69 (31.4%) reported pain in three locations, 61 (27.7%) reported pain in four locations, and 7 (3.2%) reported pain in five locations (Table 2).

**Table 2:** Number of painful body locations and the most painful body location by gender and instrument type.

Number of painful body locations	Male	Female	Strings		Woodwinds	Brass	Percussion
			Upper	Lower			
1	13	2	5	3	3	3	1
2	45	23	19	12	17	8	12
3	15	54	21	18	15	12	3
4	6	55	28	14	10	8	1
5	1	6	5	1	1	0	0

Most painful body locations	Male	Female	Strings		Woodwinds	Brass	Percussion
			Upper	Lower			
Neck	15	28	31	6	5	0	1
Left shoulder and upper arm	6	25	19	4	5	2	1
Right shoulder and upper arm	10	18	5	20	0	2	1
Right hand and wrist	13	12	3	0	14	8	0
Left forearm and elbow	13	10	8	3	5	0	7
Lower back	6	16	3	12	0	7	0
Right forearm and elbow	4	15	3	2	5	3	6
Left hand and wrist	6	10	6	1	6	2	1
Face and lips	7	6	0	0	6	7	0

For those who reported pain in only one body location (*n* = 15), the majority (*n* = 13) were male. Assessed by instrument type, these musicians were primarily string players, including five upper-string and three lower-string players, followed by three woodwind players, three brass players, and one percussionist. Among musicians reporting pain in two body locations (*n* = 68), males (*n* = 45) outnumbered females (*n* = 23). String players again constituted the largest proportion, including 19 upper-string and 12 lower-string players. This was followed by woodwind players (*n* = 17), percussionists (*n* = 12), and brass players (*n* = 8). Musicians experiencing pain in three body locations (*n* = 69) were predominantly female (*n* = 54). Within this group, string players remained the largest subgroup, including 21 upper-string and 18 lower-string players, followed by woodwinds (*n* = 15), brass (*n* = 12), and percussion (*n* = 3). For musicians reporting pain in four body locations (*n* = 61), the majority were female (*n* = 55), with only six males. String players again represented the largest group, including 28 upper-string and 14 lower-string players. The remaining participants comprised woodwind players (*n* = 10), brass players (*n* = 8), and a single percussionist. Finally, among the musicians who reported pain in five body locations (*n* = 7), six were female and one was male. Most were string players, including five upper-string and one lower-string musician, with one woodwind player.

As shown in Table 2, the neck was reported as the most painful body location among orchestra musicians (*n* = 43), including 15 males and 28 females; the majority were string players (*n* = 37), with 31 from upper strings and six from lower strings, along with five woodwind and one percussion player. This was followed by the left shoulder and upper arm (*n* = 31), reported by six males and 25 females, predominantly string players (*n* = 23, comprising 19 upper and four lower strings), and including five woodwind, two brass, and one percussion player. The right shoulder and upper arm were reported by 28 musicians (10 males and 18 females), mostly lower string players (*n* = 20), along with five upper string players, two brass and one percussion player. Pain in the right hand and wrist was reported by 25 musicians (13 males and 12 females), with the largest group being woodwind players (*n* = 14), followed by brass (*n* = 8) and three upper string players. The left forearm and elbow were cited by 23 musicians (13 males and 10 females), including 11 string players (eight upper, three lower), seven percussion and five woodwind players. Lower back pain was reported by 22 musicians (six males and 16 females), with 12 lower string, seven brass, and three upper string players. Other commonly reported painful locations included the right forearm and elbow (*n* = 19; four males, 15 females), distributed across percussion (*n* = 6), strings (*n* = 5, with three upper and two lower), woodwind (*n* = 5), and brass (*n* = 3); the left hand and wrist (*n* = 16; six males, 10 females), involving strings (*n* = 7, with six upper and one lower), woodwind (*n* = 6), brass (*n* = 2), and percussion (*n* = 1); and the face and lips (*n* = 13; seven males, six females), all of whom were woodwind (*n* = 6) or brass (*n* = 7) players. Overall, female musicians reported higher frequencies for the most painful body locations compared to their male counterparts, except for the right hand and wrist, left forearm and elbow, and face and lips; in these locations, male counts were similar or slightly higher. Instrument-specific patterns were evident, with upper string players predominating for the neck and left upper limbs; lower string players predominating for the right upper limbs and lower back; woodwind players predominating for the right hand, wrist, face, and lips; brass players predominating for the lower back, face and lips; and percussion players predominating for the left and right forearm and elbow.

### Comparative analysis: characteristics with and without PRMDs

Table 3 summarizes the characteristics of the participants when divided into PRMD and non-PRMD groups. Due to the non-normal distribution of the continuous variables, Mann–Whitney U tests were conducted to compare the groups. Statistically significant differences were observed between the groups in terms of age (Z = −9.670, *p* < 0.001) and years of playing their instrument (Z = −2.005, *p* = 0.045), with those in the PRMD group being nearly four years younger on average, and with fewer playing years than those in the non-PRMD group.

**Table 3:** Participants’ characteristics of PRMDs and non-PRMDs.

Variables	PRMDs	Non-PRMDs	Z*	χ^2^**	*p*-value
Gender, Male:Female (n)	80:140	191:113		35.801	**< 0.001**
Instrument type, strings: woodwinds: brass: percussion (n)	126:46:31:17	112:79:69:44		23.053	**< 0.001**
Employment status, full-time:part-time (n)	124:96	170:134		0.010	0.920
Age	35 (33, 38)	39 (36, 42)	−9.670		**< 0.001**
Years in orchestra	19 (15, 23)	19 (15, 24)	−0.536		0.592
Weekly playing hours (inside orchestra)	19 (16, 22)	19 (16, 23)	−1.450		0.147
Weekly playing hours (outside orchestra)	9 (7, 10)	9 (7, 11)	−1.180		0.238
Years of playing instrument	21 (18, 23)	21 (18, 24)	−2.005		**0.045**

*Mann–Whitney U test for continuous data not normally distributed. **Chi-squared test for categorical variables. ***Values in bold are statistically significant.

A significant association was also found between gender and PRMDs (χ^2^ = 35.801, *p* < 0.001). The proportion of female musicians was notably higher in the PRMD group (63.6%), whereas male musicians constituted a larger proportion of the non-PRMD group (62.8%). Similarly, instrument type exhibited a significant association with PRMDs (χ^2^ = 23.053, *p* < 0.001). String players represented a substantial proportion of the PRMD group (57.3%). In contrast, woodwind (20.9%), brass (14.1%), and percussion (7.7%) players had relatively lower representation in the PRMD group, indicating that playing string instruments may be associated with a higher prevalence of PRMDs.

### Pain intensity and pain interference scores

Within the PRMD group (*n* = 220), both pain intensity (W = 0.965, *p* < 0.001) and pain interference (W = 0.985, *p* = 0.023) significantly deviated from a normal distribution. Therefore, these variables are presented as median with interquartile range (IQR). The median pain intensity score was 12.0 (IQR: 11.0–13.0; range 0–40), and the median pain interference score was 15.0 (IQR: 13.0–17.0; range 0–50). For comparison with previous studies that reported mean scores, the mean ± SD values were 12.19 ± 1.916 for pain intensity and 15.26 ± 3.214 for pain interference.

Spearman’s rank correlation analysis was conducted to examine associations between continuous variables and pain outcomes. No statistically significant correlations were found between any of the continuous variables and pain intensity or pain interference (all *p* > 0.05). For pain intensity, the correlation coefficients were: age (r_s_ = −0.005, 95% CI: −0.135 to 0.136, *p* = 0.942), years of playing experience (r^s^ = −0.057, 95% CI: −0.183 to 0.065, *p* = 0.400), years in orchestra (r^s^ = −0.055, 95% CI: −0.191 to 0.078, *p* = 0.414), weekly playing hours inside the orchestra (r^s^ = −0.061, 95% CI: −0.192 to 0.064, *p* = 0.370), and weekly playing hours outside the orchestra (r^s^ = 0.001, 95% CI: −0.132 to 0.134, *p* = 0.991). For pain interference, the correlation coefficients were: age (r^s^ = −0.073, 95% CI: −0.209 to 0.069, *p* = 0.283), years of playing experience (r^s^ = −0.013, 95% CI: −0.146 to 0.117, *p* = 0.852), years of orchestra membership (r^s^ = −0.093, 95% CI: −0.220 to 0.037, *p* = 0.170), weekly playing hours inside the orchestra (r^s^ = 0.063, 95% CI: −0.073 to 0.201, *p* = 0.352), and weekly playing hours outside the orchestra (r^s^ = −0.051, 95% CI: −0.193 to 0.071, *p* = 0.452).

For categorical variables, Mann–Whitney U tests (for gender and employment status) and Kruskal-Wallis tests (for instrument type) were conducted to compare pain intensity and pain interference scores across groups. Specifically, no significant difference in pain intensity was found between female and male musicians (Z = −1.540, *p* = 0.124), nor in pain interference (Z = −0.785, *p* = 0.433). Across instrument types, Kruskal-Wallis tests revealed no significant differences in pain intensity (H(3) = 0.534, *p* = 0.911) or pain interference (H(3) = 0.576, *p* = 0.902). For employment status, full-time and part-time musicians showed no significant differences in pain intensity (Z = −0.244, *p* = 0.808) or pain interference (Z = −0.115, *p* = 0.909). The effect sizes were small across all comparisons, indicating negligible differences between groups. As shown in Table 4.

**Table 4:** Pain intensity and pain interference.

Categorical variables		Pain intensity				Pain interference			
		Median (IQR)	Effect size	Test statistic	*p*-value	Median (IQR)	Effect size	Test statistic	*p*-value
Gender	Female	12 (11, 13)	0.104	Z = −1.540	0.124	15 (13, 17)	0.053	−0.785	0.433
	Male	12 (11, 13)				15 (13, 17)			
Instrument type	Strings	12 (11, 13)		H (3) = 0.534	0.911	15 (13, 17)		H (3) = 0.576	0.902
	Percussion	12 (11, 14)				15 (14, 16)			
	Woodwinds	12 (11, 13)				15.5 (14, 17)			
	Brass	13 (11, 13)				15 (12.5, 17.5)			
Employment status	Full-time	12 (11, 13)	0.016	Z = −0.244	0.808	15 (13, 17)	0.008	Z = −0.115	0.909
	Part-time	12 (11, 13)				15 (13, 17)			

Continuous variables	Pain intensity			Pain interference		
	r_s_	95% CI	*p*-value	r_s_	95% CI	*p*-value
Age	−0.005	(−0.135, 0.136)	0.942	−0.073	(−0.209, 0.069)	0.283
Years of playing instrument	−0.057	(−0.183, 0.065)	0.400	−0.013	(−0.146, 0.117)	0.852
Years in orchestra	−0.055	(−0.191, 0.078)	0.414	−0.093	(−0.220, 0.037)	0.170
Weekly playing hours (inside orchestra)	−0.061	(−0.192, 0.064)	0.370	0.063	(−0.073, 0.201)	0.352
Weekly playing hours (outside orchestra)	0.001	(−0.132, 0.134)	0.991	−0.051	(−0.193, 0.071)	0.452

Effect size (r) is reported for Mann–Whitney U tests only.

### Associated factors

Univariate binary logistic regression analysis was conducted to examine the association between potential associated factors and PRMD occurrence among orchestra musicians (Table 5). Age was significantly associated with PRMDs (*β* = −0.208, OR = 0.812, 95% CI: 0.775–0.851, *p* < 0.001), indicating that younger musicians were more likely to report PRMDs. Similarly, years of playing their instrument was significantly associated with PRMDs (*β* = −0.056, OR = 0.946, 95% CI: 0.904–0.989, *p* = 0.015). Among categorical variables, gender demonstrated a significant relationship, with female musicians having a higher likelihood of experiencing PRMDs compared to males (*β* = 1.085, OR = 2.958, 95% CI: 2.064–4.240, *p* < 0.001). In addition, instrument group showed a significant association with PRMDs, particularly among string players, who had a higher risk compared with percussion players (*β* = 1.069, OR = 2.912, 95% CI: 1.574–5.385, *p* < 0.001).

**Table 5:** Univariable and multivariable analysis of associated factors.

Variables		Univariable analysis				Multivariable analysis			
		β	OR	OR 95% CI	*p*-value	β	OR	OR 95% CI	*p*-value
Age		−0.208	0.812	0.775, 0.851	**< 0.001**	−0.214	0.807	0.768, 0.849	**< 0.001**
Years of playing instrument		−0.056	0.946	0.904, 0.989	**0.015**	−0.001	0.999	0.945, 1.055	0.963
Years in orchestra		−0.010	0.990	0.956, 1.025	0.575				
Weekly playing hours (inside orchestra)		−0.037	0.964	0.921, 1.008	**0.107**	−0.034	0.966	0.917, 1.019	0.207
Weekly playing hours (outside orchestra)		−0.054	0.947	0.876, 1.025	**0.179**	−0.055	0.947	0.863, 1.039	0.249
Gender	Male (Ref)								
	Female	1.085	2.958	2.064, 4.240	**< 0.001**	1.115	3.051	2.023, 4.602	**< 0.001**
Instrument type	Strings	1.069	2.912	1.574, 5.385	**< 0.001**	1.047	2.850	1.423, 5.706	**0.003**
	Woodwinds	0.410	1.507	0.773, 2.938	0.228	0.421	1.523	0.716, 3.239	0.274
	Brass	0.151	1.163	0.576, 2.346	0.674	0.160	1.174	0.531, 2.597	0.692
	Percussion (Ref)								
Employment status	Full-time	0.018	1.018	0.718, 1.444	0.920				
	Part-time (Ref)								

Bold values indicate statistically significant results (*p* < 0.05).

Other variables—including years in orchestra, weekly playing hours inside and outside of the orchestra, and employment status—were not significantly associated with PRMDs (all *p* > 0.05).

To identify independent associated factors of PRMDs and control for potential confounding effects, variables with a *p*-value < 0.25 in the univariate logistic regression analysis were selected for inclusion in a subsequent multivariate binary logistic regression model. This approach allows potentially important variables to be retained in the analysis, even if they do not reach the conventional significance level of *p* < 0.05 in the univariate analysis; this improves the robustness of the final model. Based on this criterion, age, years of playing an instrument, weekly playing hours inside the orchestra, weekly playing hours outside the orchestra, gender, and instrument group were included in the multivariate logistic regression analysis. Before multivariate logistic regression, multicollinearity among these variables were assessed using variance inflation factors (VIF). All VIF values were below 5, indicating no multicollinearity. The Hosmer-Lemeshow test indicated good model fit (χ^2^ = 9.584, df = 8, *p* = 0.295). The Cox & Snell R^2^ and Nagelkerke R^2^ were 0.253 and 0.340, respectively. In this model, age remained an associated factor of PRMDs (*β* = −0.214, OR = 0.807, 95% CI: 0.768–0.849, *p* < 0.001), suggesting that younger musicians were more likely to experience PRMDs. In addition, gender was significantly associated with PRMDs, with female musicians having approximately three times higher odds of reporting PRMDs compared with their male counterparts (β = 1.115, OR = 3.051, 95% CI: 2.023–4.602, *p* < 0.001).

Instrument type was also identified as an associated factor, specifically for string players, who showed a significantly higher association of PRMDs compared with percussion players (β = 1.047, OR = 2.850, 95% CI: 1.423–5.706, *p* = 0.003).

In contrast, years of playing an instrument, weekly playing hours inside the orchestra, and weekly playing hours outside the orchestra were not significantly associated with PRMDs in the multivariate model (each *p* > 0.05). Similarly, woodwind and brass players did not show statistically significant associations with PRMDs when compared with percussion players.

## Discussion

### Lifetime, 12-month, 1-month, and point prevalence

The overall questionnaire response rate (51%) was consistent with several studies carried out with professional orchestra musicians (22, 27–30). In this study, the lifetime prevalence of PRMDs was high, at 84.5% (*n* = 443), indicating the widespread occurrence of PRMDs among Malaysian orchestra musicians. This result aligns closely with some international studies. This high degree of consistency is likely attributable to the use of the same operational definition of PRMDs (21) and the same assessment instrument (MPIIQM) across these studies, which enhances cross-study comparability. For example, a recent cross-sectional study among professional orchestra musicians in Poland reported a lifetime prevalence of 86.8% using the Polish version of the MPIIQM (MPIIQM-P) (23). Similarly, professional orchestra musicians in South Africa showed a lifetime prevalence of 76% (19), and a Scottish study using the original MPIIQM reported 77.2% (22). The consistently high lifetime prevalence reported across different countries—assessed with the same operational definition and assessment tool—highlights the global burden of PRMDs among professional orchestral musicians.

This study’s 12-month prevalence of 48.1% (*n* = 252) is also consistent with the samples from Poland (45.6%; 12-month prevalence using MPIIQM-P) (23) and South Africa (49%; 12-month prevalence using MPIIQM) (19) which adopted the same definition with this study. In contrast, studies employing broader definitions report higher 12-month prevalence, such as the 52% in Swedish orchestral musicians (31) and over 80% in Danish musicians (32). The prevalence rate appears dependent on the operational definition, with a narrower definition resulting in a more conservative rate, indicating that more accurate cross-research comparisons are needed.

At the 1-month level, the prevalence in the Malaysian sample (40.6%, *n* = 213) also aligns with the figures of 39.0% from Poland (23) and 30% from South Africa (19). Surveys with broader definitions often yield high 1-month or short-term prevalence estimates. For example, in a large cross-sectional study of Finnish professional symphony orchestra musicians, a large proportion of participants reported musculoskeletal symptoms affecting the back (39%), neck (69% of females, 52% of males), shoulder (21%), elbow (14%), wrist (14%), and fingers (13%) over the previous month, based on general musculoskeletal symptom questionnaires (33). Similarly, studies using broader pain definitions, such as those by Steinmetz et al. (30), found that 62.7% of professional orchestra musicians reported experiencing musculoskeletal pain in the previous 3 months.

The point prevalence of 39.5% (*n* = 207) in this study is similarly aligned with point estimates from studies using MPIIQM, such as 30% in South Africa (19) and 36.6% in Scotland (22). Again, studies with broader definitions show higher current or point prevalence, ranging to over 60% (28, 31, 34).

From the above findings, although the prevalence varies depending on the operational definition and assessment tool used, a large proportion of professional orchestral musicians may experience PRMD symptoms during their careers. The similarities in PRMD patterns between Malaysian and Western orchestras may be explained by comparable occupational demands across different cultural contexts. Globally, symphony orchestras share similar instrument configurations, sectional hierarchies, and playing techniques (32). As a result, musicians from different types of orchestras show high similarity in the nature, characteristics, and prevalence of PRMDs (35). These findings suggest that biomechanical and psychosocial exposures associated with orchestral performance are largely comparable across geographical regions. These findings reinforce the need for future epidemiological research to use standardized operational definitions and assessment tools to enable meaningful international comparisons (6, 11–15).

In the literature, approaches to defining PRMDs can be distinguished. The broadest approach relies on pain-only measures, in which PRMDs are identified based solely on the presence of pain or musculoskeletal symptoms, without requiring functional impact (31, 32). A more specific and widely accepted approach is the functional interference definition proposed by Zaza & Farewell (21), which distinguishes clinically and occupationally relevant symptoms from transient or non-disabling discomfort and is widely regarded as the standard in performing arts medicine. The MPIIQM (16) was selected in this study because it operationalizes this functional interference definition within a validated instrument for orchestral musicians, while also capturing pain intensity and interference within a biopsychosocial framework. This operational choice has important implications for comparability. It enhances consistency with studies using the same definition, supporting valid cross-study comparisons, but limits direct comparability with pain-only studies, which typically yield higher prevalence estimates due to inclusion of non-disabling symptoms. Therefore, comparisons are restricted to methodologically comparable studies, and differences in operational definitions are considered when interpreting prevalence estimates.

### Characteristics of pain locations by gender and instrument types

In this study, the neck, shoulders, and upper limbs were the most commonly cited body locations for pain by orchestra musicians. Other international literature also describes distributions including the neck, shoulder, and upper limb complex as the most affected by PRMDs across different orchestra groups and methods (13, 16, 22, 28–30, 32, 34, 36–38). Various studies indicate that upper-body vulnerability is a common phenomenon for orchestra musicians.

High multi-site pain prevalence has been reported in orchestra musicians from Brazil [55%; (28)], Germany [43%; (30)], and Scotland [43%; (22)]. In this study, 62.3% (*n* = 137) of participants reported pain in three or more areas, indicating multiple sites of injury resulting from performance. In general, this shows that PRMDs in professional orchestral musicians are rarely isolated, single-point conditions, but are often a cumulative and distributed burden involving multiple anatomical areas. However, the reported multi-site variability may also be caused by different PRMD recall periods and operational definitions.

PRMD patterns were strongly instrument-specific in this study. Among upper-string players, symptoms in the neck, left shoulder, and left upper limb predominated; this is consistent with previous studies describing elevated left-sided cervical and shoulder loading associated with sustained asymmetric postures, prolonged arm elevation, and static neck rotation during violin and viola performance (13, 19, 29, 30, 36). Higher cumulative exposure arising from early training onset, extensive daily practice hours, and greater repertoire-related workload in upper-string players has been proposed as contributing to the burden observed in this group (19, 34, 39). Biomechanical research further demonstrates that sustained activation of the trapezius, levator scapulae, and rotator cuff musculature during prolonged asymmetric playing postures can intensify these symptoms (14).

Most lower-string players reported pain in the right shoulder, right upper limb, and lower back, consistent with bowing-arm loads. Electromyography studies show that the deltoid muscle and superior spinous muscles are relatively active when playing in the loud kinematics and upper range (40). Movement analysis shows that the upper arm is raised during high-pitched cello performance, which increases the shoulder force. However, not all biomechanical evidence is consistent. Nyman et al. (39) propose that the relatively neutral arm posture adopted by cellists provides protection against neck and shoulder symptoms. This difference may indicate a complex interrelationship between individual technique, repertoire demands, and individual adaptation.

Findings among woodwind players in this study indicated involvement of the right hand, wrist, face, and lips. This aligns with prior literature that highlights thumb and hand symptoms associated with prolonged instrument support among oboists and clarinetists (41). However, some studies have reported frequent elevated left shoulder and neck symptoms among flautists due to sustained arm elevation and the transverse holding posture (22, 34, 39, 41). This further indicates that personal technical variation, compensatory posture adjustment, or sampling instability may contribute to such outcomes.

Findings among brass players indicated involvement of the lower back, face, and lips. The lower back symptoms are consistent with previous studies, which have reported that brass performance requires sustained trunk stabilization, support of the instrument, and maintenance of controlled breathing (42, 43). Similarly, the involvement of the face and lips is in line with the demands of brass playing. Sound production relies on embouchure control, which requires continuous activation of facial muscles and sustained pressure of the mouthpiece against the lips (42, 43). However, the relatively less involvement of the upper limbs in the present study contrasts with previous literature, in which the shoulders, arms, and hands are reported as common sites of PRMDs among brass players (42, 43). This discrepancy may be related to differences in sample characteristics, playing contexts, or instrument distribution among participants.

Percussionists reported that their PRMD manifested as bilateral forearm and elbow symptoms. A study on active Greek professional percussionists found that the highest proportion of respondents had issues with the upper limbs (32%), including neuropathy symptoms, and spine or back pain (20%) (44). In comparison, no participants reported back pain in this study. This may be due to the small number of percussionists in our sample, and should be interpreted cautiously.

Previous studies have shown that the prevalence of PRMDs is higher among female musicians, particularly in the neck, shoulders, and upper limbs (22, 29, 30, 32). Consistent with these findings, we also observed a higher occurrence of PRMD symptoms among female musicians in these body areas.

In general, the locations of PRMDs observed in this study were generally similar to those reported in international studies. Differences between instrument groups and genders may be explained by methodological heterogeneity, subgroup sample limitations, and contextual factors. Future studies should make use of standardized measurements and detailed exposure characterization to improve risk analysis and develop targeted prevention strategies.

### Associated factors for PRMDs

This study identified female gender, playing string instruments, and younger age as significant associated factors for PRMDs among orchestra musicians.

The high risk observed in female musicians is consistent with several large-scale epidemiological surveys. Paarup et al. (32) reported a significant increase in the prevalence and symptoms of PRMDs in female orchestra musicians; similarly, Steinmetz et al. (30), Leaver et al. (29), and Berque et al. (22) all identified female gender as a significant associated variable of PRMDs in European orchestral contexts. Australian professional samples (37) and earlier studies (21) also report the same trend. Proposed mechanisms include increased relative biomechanical load due to low absolute muscle strength, differences in gender-related pain perception and reporting behaviors, and increased exposure to psychosocial stressors related to performance needs (14, 32). However, some studies did not find statistically significant gender differences, although these results were from smaller or methodologically heterogeneous samples (28, 31). The differences in results may be due to insufficient statistical capacity, variation in definitions, or differences in measurements.

The higher prevalence of PRMDs among string players in this study is highly consistent with previous literature. Compared with woodwind and brass players, violinists and other string musicians consistently show a higher prevalence and greater severity of PRMDs, particularly involving the neck, shoulders, and upper limbs (13, 19, 21, 29, 30, 34, 36). This pattern is thought to be associated with sustained asymmetric postures, repetitive fine motor demands, and high cumulative playing exposure in string performance (14, 39). Electromyographic and kinematic studies have also found increased activation of cervical and shoulder muscles during intensive string playing, suggesting a biomechanical link with symptom development (40). However, some studies have reported relatively weak or insignificant differences between instrument groups, particularly in mixed samples or when broad, non-operational definitions are applied (28, 31). This highlights the importance of sampling structure and operational definition for cross-study comparability.

Young musicians have an elevated risk of PRMDs. Accumulated experience may improve motor efficiency, physical awareness, preventative strategies, and self-management ability, thus reducing the risk of injury over time (14, 21, 30, 45). Fishbein et al. (27) similarly propose that the prevalence of PRMDs slowly declines after reaching its peak in early to mid-adulthood, related to adaptive physiological and behavioral changes. However, contradictions exist in literature. Berque et al. (22) found an increased risk of PRMDs in older and more professionally experienced musicians, while other reports show no obvious link between age and pain outcomes (28, 46). Kenny & Ackermann (35) proposed a further nonlinear model, which found that musicians in the 40 to 50 years old age group suffered more than younger or older people. These inconsistencies likely arise from differences in sampling structures, age classification, exposure duration, occupational sustainability, and potential survivor effects; musicians with severe PRMDs may exit professional performance earlier. Therefore, whether age is associated with PRMDs requires more rigorous discussion.

Overall, the convergence of female gender, string instrument performance, and younger age as associated factors in this study reinforces the view that PRMDs arise from interacting mechanisms, rather than single isolated determinants. Targeted prevention strategies—sensitive to demographic characteristics, instrument-specific demands, and career stage—are therefore needed.

### Pain intensity and pain interference

Musicians with PRMDs in this study reported a mean pain intensity score of 12.19 ± 1.916 (range 0–40) and a mean pain interference score of 15.26 ± 3.214 (range 0–50). These values are highly comparable to those observed in professional orchestral musicians by Berque et al. (22), who reported mean pain intensity and interference scores of 12.4 ± 7.63 and 15.2 ± 12.39, respectively, using the MPIIQM and the same operational definition. Slightly higher scores have been reported in South African orchestral musicians (intensity: 16.3 ± 6.62; interference: 21.2 ± 9.98) (19) and Portuguese professional musicians (intensity: 14.99 ± 3.75; interference: 17.45 ± 3.49) (20), both of which also used the MPIIQM with the same definition.

Evidence from studies using numeric rating scales suggests that PRMD-related pain is typically intermittent and of mild-to-moderate severity rather than acutely disabling (30, 37). However, direct comparison with these studies should be interpreted with caution due to differences in measurement instruments and operational definitions. This highlights the importance of standardized pain measurement approaches in performing arts medicine research to improve cross-study comparability.

With respect to gender differences, the extensive literature on PRMDs indicates that female participants generally report higher pain prevalence, greater pain sensitivity, and higher levels of pain-related disability; this may reflect biological differences in nociceptive processing as well as psychosocial and occupational influences (47–49). In this study, female musicians reported higher mean pain intensity and interference scores than their male counterparts. However, these differences were not statistically significant. This lack of effect may reflect limited statistical power or personal variability in pain reporting. Future studies will require larger samples and strict measurement approaches to clarify whether sex-related differences encompass pain severity and functional interference among orchestral musicians.

### Limitations

A limitation of this study relates to the operational definition of PRMDs, which was based on functional interference rather than explicit attribution of symptoms to playing-related causes. Consequently, some non-playing-related musculoskeletal symptoms may have been included, potentially leading to an overestimation of PRMD prevalence. Future studies should consider instruments that better distinguish playing-related from non-playing-related symptoms.

A second limitation is the cross-sectional design, which precludes causal inference. In addition, several psychosocial and workload-related factors identified in recent longitudinal and multicenter studies were not included in this analysis. This may have introduced residual confounding, and future longitudinal studies incorporating multidimensional risk factor models are needed to better clarify causal pathways.

A third limitation concerns the sampling strategy. The use of convenience sampling may limit generalizability to the wider population of orchestral musicians in Malaysia. Self-selection bias and potential survivor bias may also be present, as musicians with more severe symptoms may have reduced participation or exited professional performance. In addition, the overrepresentation of string players may have influenced prevalence estimates and associated patterns.

Finally, the inclusion criterion of a minimum of 10 years of playing experience may have introduced selection bias by excluding early-career musicians and limiting variability in exposure history. While this criterion was used as a pragmatic proxy for cumulative occupational exposure, it may reduce the generalizability of findings to musicians with more diverse career stages.

## Conclusion

This study provides the first epidemiological evidence relating to PRMDs among orchestra musicians in Malaysia. PRMDs are relatively common, generally involve multiple body locations, and are associated with moderate pain intensity and interference; our results indicated a substantial occupational health burden, but one unlikely to cause extensive work disruption. Female musicians and string players were identified as groups more likely to report PRMDs, whereas older age appeared to confer a protective effect. The use of a standardized measurement tool, the MPIIQM, to obtain these findings allowed for meaningful comparisons with international literature and enrichment of the evidence base of performing arts medicine. Future research should combine longitudinal and multi-cultural studies, integrating high-quality assessment tools, to determine causal mechanisms and evaluate the effectiveness of preventive strategies.

## Data Availability

The raw data supporting the conclusions of this article will be made available by the authors, without undue reservation.
